# Plasticized Starch/Gelatin Blends with Humidity-Activated Shape-Memory Behavior

**DOI:** 10.3390/polym17131763

**Published:** 2025-06-26

**Authors:** Victor Oliver-Cuenca, Ana Muñoz-Menzinger, Marina P. Arrieta, Daniel López, Laura Peponi

**Affiliations:** 1 Instituto de Ciencia y Tecnología de Polímeros, ICTP-CSIC, Calle Juan de la Cierva 3, 28006 Madrid, Spain; victor.oc@ictp.csic.es (V.O.-C.); anamenzing@ictp.csic.es (A.M.-M.); daniel.l.g@csic.es (D.L.); 2 Departamento de Ingeniería Química Industrial y del Medio Ambiente, Escuela Técnica Superior de Ingenieros Industriales, Universidad Politécnica de Madrid (ETSII-UPM), Calle José Gutiérrez Abascal 2, 28006 Madrid, Spain; m.arrieta@upm.es; 3 Grupo de Investigación: Polímeros, Caracterización y Aplicaciones (POLCA), 28006 Madrid, Spain

**Keywords:** starch, gelatin, shape-memory, humidity

## Abstract

Biodegradable and bio-based polymers, such as starch and gelatin, are emerging as an important alternative to the use of conventional polymers. In this work, different proportions (1/1, 1/1.5, 1/2, and 1/2.5) of these bio-based polymers will be investigated, with the primary objective of considering their strong moisture dependence as an advantage instead of a problem, as commonly considered. For this interesting challenge, the humidity-activated shape memory effect has been studied in both neat and plasticized starch. Additionally, for the first time, to the best of our knowledge, the shape-memory behavior activated by humidity in gelatin, as well as in starch/gelatin blends, is reported. In all cases, starch, gelatin, and their plasticized blends show excellent values in terms of strain fixity ratio, obtaining values of about 100% in all cases, and strain recovery ratio, with values higher than 90% for the samples studied. Moreover, considering their potential application as food packaging, mechanical response, wettability, water permeability, water uptake rate, and roughness is also studied in this work, taking into account the effect of the different amounts of gelatin on the final behavior of the materials.

## 1. Introduction

Nowadays, there is a growing need to develop biodegradable materials with sustainable properties for use as polymeric matrices in various economic sectors. One of the most important is the packaging sector, which accounts for the largest share of plastic waste generation worldwide, with 40.5% by segment in the EU-27 + 3 (27 EU member states plus Switzerland, Norway, and the United Kingdom) [1]. Growth is expected to double by 2050 [2]. These plastics, mainly derived from petroleum, not only consume a non-renewable resource for their production, but also, when wrongly managed, can negatively affect the environment after their end-of-life, impacting aquatic and terrestrial soil ecosystems and, as a result, harming wildlife and human health. In light of this concerning situation, there has been a notable increase in requests for sustainable and biodegradable solutions, leading to the development of new packaging materials that can substitute conventional plastics [3]. However, bio-based polymers possess lower performance than traditional plastics. Therefore, the blending process is a widely used and relatively simple method for tailoring the physical and mechanical properties of biopolymers. In this context, the combination of thermoplastic starch and gelatin has emerged as a promising and viable alternative for developing sustainable food packaging materials.

Shape-memory polymers (SMPs) have emerged as a subject of considerable interest in both the academic and industrial fields, with a particular focus on their potential applications in the biomedical sector [4]. SMPs can retain their original shape following deformation to a temporary shape under the influence of external stimuli, such as temperature, humidity, pH, light, and so forth [5]. These shapes are induced by forcing a temporal shape during the so-called “programming” stage, followed by the recovery of the original shape, during the “recovery” stage, through the action of the external stimuli [6].

In humidity-activated shape-memory behavior, the presence of hydrogen bonds is the primary factor enabling the recovery of the original shape. Therefore, the capacity of the polymer matrix to retain water is a significant contributing factor [7].

Considering all these properties, biodegradability, hydrophilicity, and biocompatibility, starch and gelatin can be considered as suitable materials for designing humidity-activated shape-memory materials. To the best of our knowledge, no studies have been reported in the scientific literature about the humidity-activated shape-memory response of gelatin.

Starch is a natural bio-based polymer obtained from various natural sources, although the most studied ones are those extracted from potato, rice, and maize. Thus, the botanical source of starch will impact its physicochemical properties. This influence may be attributed to several factors, including the distribution of granule size, the degree and/or polymorphic form of crystallinity, the organization of molecules within the granule, and the chemical nature of the starch [8]. In general, the structure of starch consists of a sequence of α-D-glucose units linked together by α (1 → 4) glycosidic bonds [9]. Starch granules contain two distinct structural components: amylose and amylopectin. Amylose is a non-branched, linear, helical structure formed entirely by α (1 → 4) glycosidic bonds. In contrast, amylopectin is a highly branched structure composed of 24 to 30 glucose units. This branching occurs due to α (1 → 6) glycosidic bonds, which create branch points, in addition to the α (1 → 4) glycosidic bonds similar to those in amylose [10]. The ratio of these two components can fluctuate due to various factors, with the most significant being the source of starch extraction and the conditions under which the vegetable source is harvested. Generally, native starches contain between 20 and 30 percent amylose and 70 to 80 percent amylopectin [11].

When starch is heated in the presence of water, the structure of the granules is disrupted and transformed into a continuous, entangled polymer matrix. This process is called gelatinization of starch, resulting in thermoplastic starch [12]. Due to this process, the hydrogen bonds between the polymer chains are disrupted, allowing water molecules to intercalate between them, resulting in a malleable material that can be molded and extruded [13]. Therefore, thermoplastic starch offers many advantages as a bio-based polymer that is biodegradable under composting conditions [14]. However, its mechanical properties are somewhat limited, particularly its tensile strength and performance in environments with high humidity, as well as its poor barrier properties [15,16]. For these reasons, its application in the packaging sector is restricted. Nevertheless, despite these limitations, thermoplastic starch remains an intriguing material due to its low cost and high bioavailability.

Gelatin is a protein obtained from the partial hydrolysis of collagen found in animal bones and skins. Two distinct types of gelatins, referred to as Type I, with an isoelectric point (pI = 8–9), and Type II, with an isoelectric point (pI = 4–5), can be produced through the acidic or basic hydrolysis of collagen, respectively [17]. Furthermore, by controlling the temperature during processing, the triple helix collagen can be broken down into gelatins of varying molecular weights, resulting in gelatins with different mechanical strengths. This biopolymer features a unique structure that can enable it to form films. Gelatin-based films are translucent and offer improved tensile elasticity, making them suitable for food packaging applications. [18]. Despite the encouraging attributes of this material for its use as a packaging material, several challenges remain open before it can be adopted on an industrial scale [19]. One significant issue is the stability of the material in high-moisture environments, which could potentially compromise the integrity of the packaging, affecting the quality of the food it holds [20]. Furthermore, production cost plays a crucial role in determining the commercial viability of these materials. While biopolymers provide significant environmental benefits, they are often more expensive than conventional plastics [21]. Optimizing production processes and researching new blends of starch and gelatin can significantly help in reducing costs and making these material alternatives more competitive in the marketplace. In particular, in this work, the main objective is the study of the strong moisture effect on both starch and gelatin as a potential advantage for both neat materials as well as their blends. It is well known that starch presents humidity-activated shape-memory response [7]; however, to the best of our knowledge, no studies on humidity-activated shape-memory response on gelatin have been reported in the scientific literature. Therefore, the present study seeks to examine the humidity-activated shape-memory response of neat gelatin as well as plasticized starch/gelatin blends. Different plasticized starch/gelatin blends with different percentages % increasing the amount of gelatin, such as starch/gelatin 1/1, 1/1.5, 1/2, and 1/2.5, have been studied. In order to study the humidity-activated shape-memory effect, moisture absorption and moisture loss have also been studied, as well as their physico-chemical behavior, morphology, and mechanical response. Moreover, considering their potential application as food packaging, wettability and water permeability are also studied.

## 2. Materials and Methods

### 2.1. Materials

Native potato starch was kindly supplied by Lyckeby, Kristianstad, Sweden. Glycerol (≥99% GC) and gelatin (from porcine skin, type A, ~300 bloom) were purchased from Sigma-Aldrich, Saint Louis, MO, USA.

### 2.2. Film Preparation

Starch (S) and gelatin (G) blend films were obtained through the gelatinization of starch, gelatin, and their blends in aqueous media, with glycerol serving as a plasticizer. The films were prepared using the following method: a 1% weight/weight dispersion of the polymer in aqueous media was homogenized for 30 min for starch and 1 h for gelatin. Both dispersions were then gelatinized at 80 °C for 20 min. At the end of this period, 30 wt% of glycerol relative to the mass of the neat polymers was added to the dispersion, obtaining plasticized starch and plasticized gelatin named S-30Gly and G-30Gly, respectively. For the polymeric blends, at the initial starch dispersion, different amounts of gelatin were added and homogenized for an additional 60 min. Finally, glycerol was added. It is important to consider that the amount of glycerol is fixed at 30 wt% for starch for all the formulations, and that gelatin is added in different amounts relative to starch, such as S/G-1/1, S/G-1/1.5, S/G-1/2, and S/G-1/2.5. Therefore, the synergistic effect on the films’ properties, resulting from the interaction of starch, glycerol, and gelatin, was measured and observed. The percentages of each component in each formulation are presented in Table 1.

### 2.3. Characterization Techniques

Before characterization, all films were kept at room temperature with 40% humidity for one day.

Film thickness was measured using a digital micrometer (0.001 mm, Hangzhou United Bridge Tools Co., Ltd., Hangzhou, China).

The homogeneity of the films was measured using a field emission scanning electron microscope (PHILIPS XL30 Scanning Electron Microscope, FEI Philips, Hillsboro, OR, USA), based on the cross-section of the films obtained. The films were fractured by immersion in liquid nitrogen and subsequently cryo-fractured. Before observation by scanning electron microscopy (SEM), all samples were coated with chrome using an automatic sputter coater, model Quorum Tech Q 150T ES, UK.

A series of attenuated total reflectance-Fourier transform infrared spectroscopy (ATR-FTIR) measurements were performed using a Spectrum One FTIR spectrometer manufactured by PerkinElmer Instruments, Rodgau, Germany, in the range of 400–4000 cm^−1^. The spectrometer has a resolution of 4 cm^−1^ and four acquisitions for each spectrum. The resulting spectra were processed by using OriginPro 8.5.

Raman measurements were carried out using a confocal Raman spectrometer, Renishaw InVia Reflex Raman system, UK, coupled to a microscope. The excitation of Raman scattering is operated with a diode laser at a wavelength of 785 nm or 515 nm. The laser beam is focused on the sample by means of a ×50 microscope objective. The exposure time and accumulation for measuring the Raman spectra were 10 s and 1 time, respectively. Laser power at the sample was approximately 160 mW.

Thermogravimetric analysis (TGA) was performed using a TA-TGA Q500 thermal analyzer, Mettler Toledo, Barcelona, Spain. Different materials were analyzed in dynamic mode with a sample mass of about 10 mg. The temperature range extended from room temperature to 800 °C, with a heating rate of 10 °C/min. The analysis took place in a nitrogen atmosphere at a flow rate of 60 mL per minute. The maximum degradation temperatures (T_max_) were calculated from the first derivative of the TGA curves.

Dynamic mechanical thermal analysis (DMTA) of the samples was conducted using a DMA Q800 from TA Instruments, New Castle, DE, USA, in film tension mode with an amplitude of 5 μm, a frequency of 1 Hz, a force track of 125%, and a heating rate of 2 °C min^−1^. The samples were meticulously extracted from the cast films and subsequently fashioned into rectangular specimens with dimensions of approximately 20 mm in length and 6 mm in width.

Tensile tests were conducted at room temperature using a DX2000 QTest Elite MTS dynamometer (MTS Systems, Eden Prairie, MN, USA), equipped with a 100 N load cell and a stretching rate of 10 mm/min. Measurements for the tensile tests were carried out on dog bone specimens with a width of 2 mm.

The water permeability of the films was determined by measuring the water vapor transmission rate (WVTR) using the gravimetry method according to UNE 53097:2002. Sheet materials—Determination of water vapor transmission rate—Gravimetric (dish) method [22]. The films were placed in permeability cups with an exposed area (A) of 10 mm^2^. A quantity of 2 g of silica was introduced into the permeability caps, creating an area characterized by a relative humidity of 0% on one side of the film. The permeability cups were placed in a desiccator containing a saturated KNO_3_ solution at 20 ± 1 °C, reaching a RH ≥ 72%. In this experiment, three samples of each formulation were weighed at hourly intervals for the first 8 h, followed by a final measurement at 24 h after the start of the experiment.

For roughness measurements, a Zeta non-contact optical profilometer, model Z-20, KLA, Milpitas, CA, USA, was used at ×20 magnification. Four different regions were measured for all samples.

The surface wettability of the starch/gelatin blends was studied through static water contact angle (WCA) measurements using an Ossila goniometer device, UK. Droplets were controlled by a constant volume of 1 μL. The WCA was determined by depositing 7 random droplets onto the film surface, and the average value was reported.

Experiments measuring moisture absorption and moisture loss were conducted using a drying chamber (RH ≤ 10%) and a moisture chamber (RH ≥ 72%). For moisture absorption, dried samples were placed in the moisture chamber, and their weight was measured initially every 30 min, followed by every 60 min after the first two measurements, as the moisture time gradually increased [23,24]. The same procedure was applied to moisture loss, with the samples being placed in the drying chamber until equilibrium was reached.

The water uptake test was modified from the study by Tan et al. with slight modifications [25].

Films were cut into a size of 2 cm × 2 cm, approximately, and their mass was measured. Subsequently, the film was placed in a container containing distilled water, where it remained for a period of 24 h. During the specified period, the film was extracted from the water at 1, 2, 4, and 24 h. After the removal of excess liquid via tissue paper, the film was weighed before its reintroduction to the water.

The water uptake by the film was calculated according to Equation (1).

(1) $$Water\;uptake\;\left(\%\right)=\frac{W-W_{0}}{W_{0}}\times 100$$

Humidity/mechanical cyclical experiments were conducted using a bending test. The programming stage occurs in a dry chamber (RH ≤ 10%) at 20 °C, while the recovery stage takes place in a moisture chamber (RH ≥ 72%) at 20 °C. The samples were carefully shaped into rectangular forms with approximate dimensions of 50 mm × 10 mm × 0.1 mm.

The shape recovery ratio (*R_r_*) was determined by calculating the ratio of the various angles before and after recovery. This calculation was derived using the recovered angles (Ɵ*_r_*) and the deformed angles (Ɵ*_d_*) within the transient shape. The equation below provides the detailed formula for this calculation:

(2) $$R_{r}\left(\%\right)=\frac{\left({Ɵ}_{d}-{Ɵ}_{r}\right)}{{Ɵ}_{d}}\times 100$$

The shape fixity ratio (*R_f_*) can be calculated using the following equation:

(3) $$R_{f}\left(\%\right)=\frac{{Ɵ}_{f}}{{Ɵ}_{d}}\times 100$$

**Figure 1:**
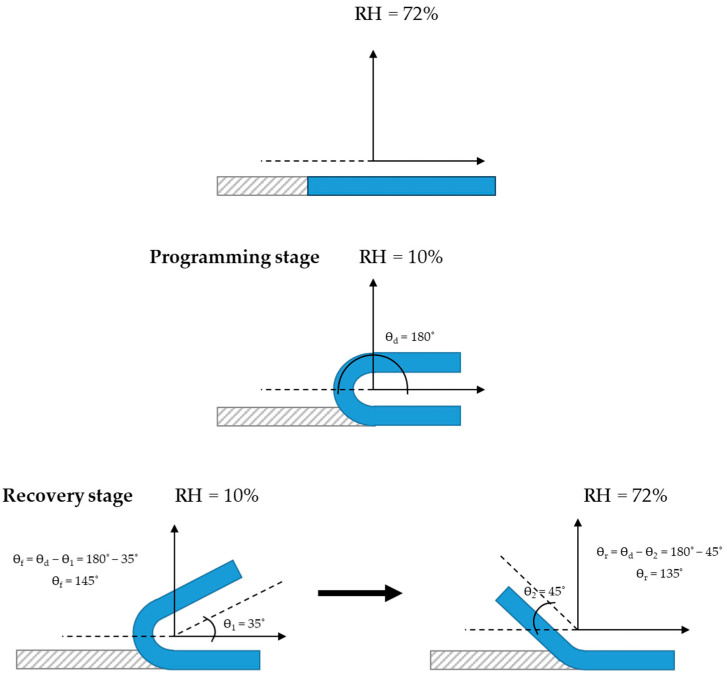
The following experiment is designed to test the bending behavior of materials in a controlled setting. The objective of this study is to examine the materials’ response to external stress, particularly in the context of their shape-memory properties.

The measurement of test specimen angles was conducted using ImageJ, v.1.54h, a publicly available image-processing software program. Figure 1 illustrates the experimental setup for studying the materials’ shape-memory behavior. Figure 1 details the angles recovered and deformed during the various stages of the programming and recovery analyses, as well as the relative humidity (RH) levels corresponding to each phase.

## 3. Results

### 3.1. Scanning Electron Microscopy (SEM)

**Figure 2:**
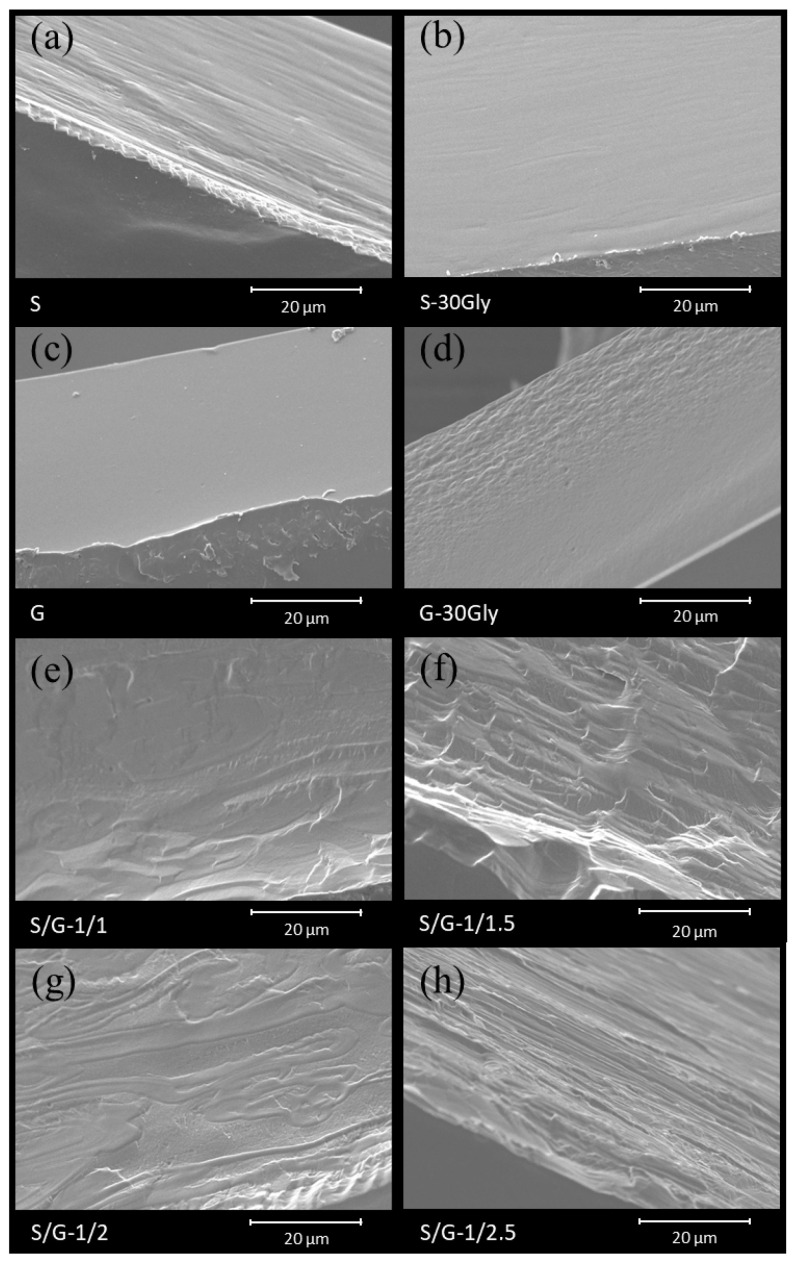
Cryo-fractured film samples observed through the scanning electron microscopy technique; (**a**) S, (**b**) S-30Gly, (**c**) G, (**d**) G-30Gly, (**e**) S/G-1/1, (**f**) S/G-1/1.5, (**g**) S/G-1/2, and (**h**) S/G-1/2.5.

The morphology of the films of the neat, plasticized, and their corresponding blends, obtained by SEM analysis conducted on a cryo-fractured cross-section of the samples, is reported in Figure 2.

The SEM images show that neat starch is homogeneous, with its homogeneity increasing through the addition of glycerol. Neat gelatin, Figure 2c, shows fine spherical structures on the surface, which may be related to the ends of collagen filaments. These filaments have clustered due to the reorganization of the helical structures that compose the polypeptide chains of scleroproteins. The SEM images of the starch/gelatin blends show structures that resemble scratches. These scratches may correlate with starch deposits on the surface of the blend as reported in the literature [26], resulting in decreased homogeneity. Moreover, a clear differentiation of the two phases can be observed in S/G-1/2, Figure 2g, and S/G-1/2.5, Figure 2h.

### 3.2. Attenuated Total Reflectance-Fourier Transform Infrared Spectroscopy (ATR-FTIR)

**Figure 3:**
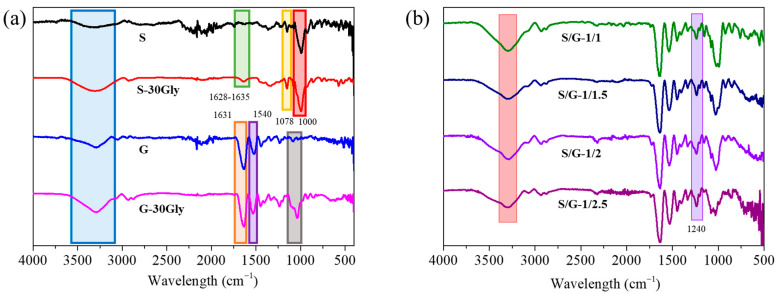
FTIR spectra of the samples (**a**) S, S-30Gly, G, and G-30Gly; (**b**) S/G-1/1, S/G-1/1.5, S/G-1/2, S/G-1/2.5. The different colors of the rectangles are used as an indicator of the different peaks observed.

The FTIR spectra of the films, as well as of the neat materials in the range from 4000 to 400 cm^−1^, are shown in Figure 3.

Starch samples present broad peaks within the range of 3236–3246 cm^−1^ associated with the stretching and vibration of the hydroxyl group. On the other hand, the peaks within the range of 1628–1636 cm^−1^ were identified as indicators of starch film swelling in response to water absorption. A peak at 1078 cm^−1^ indicated the stretching vibration of the C–O and C–O–C bonds, which is attributed to the hydrolysis of the starch films [27]. At 1000 cm^−1^, there is the major absorption band from C-O, C-C, and C-O-H stretching and C-O-H bending [27].

For gelatin, a distinct intensity peak near 3291 cm^−1^ corresponded to N–H bond stretching [28] as well as a broad peak associated with hydroxyl groups. Moreover, the peak at 1631 cm^−1^ in the amide I region of pure gelatin was assigned to the C–O vibrations of hydrogen-bonded COO^−^ groups. The peak at 1540 cm^−1^ in the amide II region of pure gelatin was attributed to the bending vibration of N–H bonds and the stretching vibrations of C–N bonds 1240 cm^−1^ [29].

Furthermore, it has been observed that a clear signal related to glycerol can be observed in the case of G-30Gly at 1300–1000 cm^−1^ corresponding to C-O functional group absorption [30], thus considering that in the rest of the materials this signal is masked.

However, in starch/gelatin blends, the peak corresponding to N-H bond stretching gets sharper as the concentration of gelatin increases. Meanwhile, no variation in the broad peak corresponding to hydroxyl groups stretching and vibration is observed. In the range from 1750 to 1300 cm^−1^, there is a superposition of the signals of both polymers. However, as the concentration of gelatin in the blends increases, the intensity of the peak at 1240 cm^−1^ increases due to the increase in the number of C-N bonds in the blends.

### 3.3. Raman Spectroscopy

**Figure 4:**
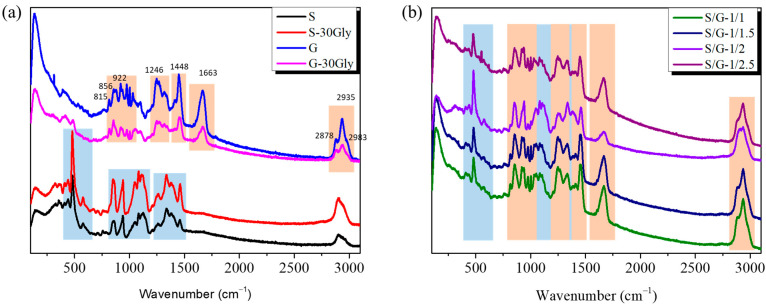
Raman spectra of the samples (**a**) S, S-30Gly, G, and G-30Gly; (**b**) S/G-1/1, S/G-1/1.5, S/G-1/2, S/G-1/2.5. Orange is used for gelatin while blue for starch bands.

In Figure 4, signals presented by neat samples, Figure 3a, and blends, Figure 3b, are shown.

For gelatin, the bands between 2800 and 3050 cm^−1^ are attributed to the C-H vibrational modes. The main bands observed on the spectra at 2878 cm^−1^ and 2935 cm^−1^, as well as the shoulder at 2983 cm^−1^, have been identified as corresponding to CH_2_ asymmetric, CH_3_ symmetric, and CH_3_ asymmetric protein bands, respectively [31].

The amide I band centered at 1663 cm^−1^ is assigned to the peptidic bond C=O stretching of the Gly-X-Y tripeptide sequence, which usually indicates the alpha helix-dominated structure [32,33]. The bands observed from 1330 to 1200 cm^−1^ correspond to the amide III vibrational modes [34], with the CH_2_-CH_3_ bending protein band at 1448 cm^−1^ [35]. The bands at 857 and 920 cm^−1^ are attributed to the proline ring C-C bond vibrations, while the 938 cm^−1^ band is ascribed to the Gly-X-Y backbone C-C stretching vibration. The C-O-C stretching band assigned to the glucosyl-galactosyl hydroxylysinonorleucine cross-link between the tropocollagens is seen at 815 cm^−1^ [34].

For starch, the bands observed at 1500–1200 cm^−1^ are predominantly attributed to the vibrations of the hydrogen-bonded carbon groups C-H, CH_2_, C-O-H, and C-C-H. The signals in question primarily reflect information concerning hydrogen bonding within the glucan chain helical structure of amylopectin and amylose [36]. The bands observed at 1200–800 cm^−1^ are attributed to vibrations in the C-C, C-O, and C-O-C stretching within glycosidic linkages and ring breathing [37]. This region contains signals derived from the α-D-(1-4) and α-D-(1-6) linkages that are specific to amylopectin and the linear glucan chains that constitute amylose. The 800–400 cm^−1^ band contains signals from C-C-C, C-C-O glycosidic ring skeletal vibrations [38].

Nevertheless, the broadening of the signal at 1663 cm^−1^ assigned to the amide I is observed, indicating the formation of hydrogen bonds between both polymers.

### 3.4. Thermogravimetric Analysis (TGA)

**Figure 5:**
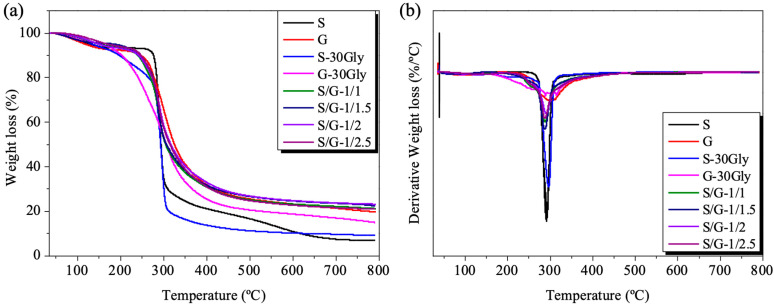
(**a**) TGA analysis of the sample evolution of the loss of weight as the temperature increases. (**b**) Derivative of the weight loss as the temperature increases.

TGA was used to study the thermal degradation of the obtained blends; see Figure 5. The films were tested for weight loss between 25 °C and 800 °C. The maximum degradation temperature, T_max_, is observed for each sample and summarized in Table 2. These blends present very high maximum degradation temperatures, considering their potential use as food packaging.

Starch samples exhibited low residue at 800 °C, while gelatin samples showed high residue (≈20%), as reported in the existing bibliography [39,40].

Nevertheless, the blends exhibited a higher level of residue compared to the neat samples. The increase in residue percentage can be attributed to the enhanced thermal stability of the protein–polysaccharide matrix, which results in the formation of thermally stable residues, as suggested in the bibliography [41].

### 3.5. Dynamic Mechanical Thermal Analysis (DMTA)

**Figure 6:**
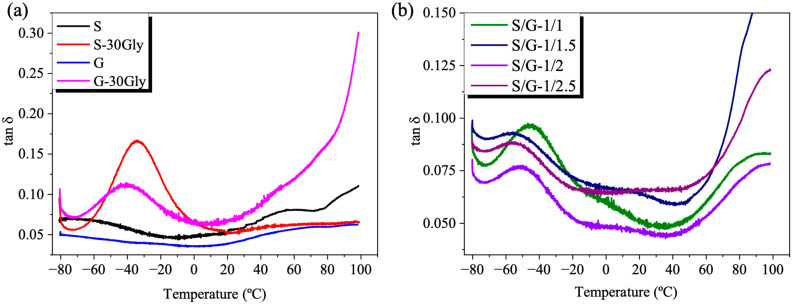
DMTA analysis for all the studied samples: (**a**) Tan δ of the neat samples, and (**b**) Tan δ of the polymeric blends.

To study the main thermo-mechanical relaxation of these systems, a DMTA was performed. The damping factor (tan δ) was measured as a function of temperature over the range of −80 °C to 100 °C. The results obtained are presented in Figure 6.

Non-plasticized samples showed only one transition (α-relaxation) corresponding to the glass transition temperature. Meanwhile, plasticized samples present two main relaxations, being the new one related to the glycerol-rich phase (β-relaxation) [42].

As shown in Figure 6, the temperatures observed for each of the main transitions are shown. As the content of gelatin increases, this relaxation becomes much weaker, as previously reported in the case of alginate and chitosan blends plasticized with glycerol [43]. This suggests that the glycerol-rich phase is strongly restricted in motion due to the network constraints.

In the blends, Figure 5b, an increase in the glass transition temperature is observed. T_g_ increased from 42° for S-30Gly and 49° for G-30Gly up to almost 90° for S/G-1/2.5. This fact is due to the presence of more interactions between the polymers.

### 3.6. Mechanical Properties

**Figure 7:**
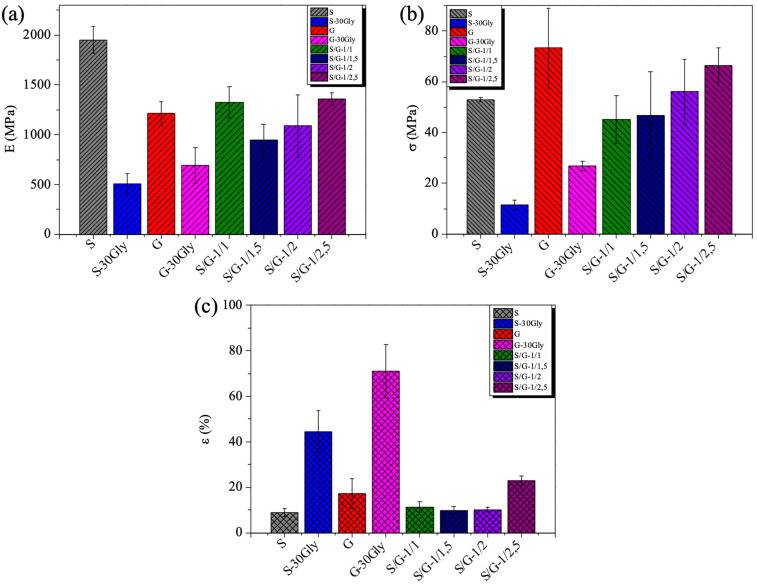
Mechanical properties of the samples: (**a**) elastic modulus (E); (**b**) tensile strength (σ); and (**c**) elongation at break (ε).

In addition to these characterization techniques, the response in terms of mechanical properties has been studied. In particular, the values for the elastic modulus, the tensile strength, and the elongation at break for both neat and plasticized materials and their blends are presented in Figure 7 and summarized in Table 3.

Neat starch film (S) showed the highest elastic modulus at 1951 MPa; this value was highly reduced in the corresponding plasticized sample by about 4 times. It is interesting to observe that the tensile strength is also reduced by about 4 times in the plasticized starch film, while the elongation at break of the plasticized starch is four times higher than the neat starch. As expected, the same effect is observed in the case of gelatin and its plasticized film, even if in this case both the elastic module and tensile strength decrease by about 2 times compared to the neat film and the elongation at break for the plasticized gelatin film is also 4 times higher than the one presented for the neat gelatin film. However, in the case of the blends, the addition of gelatin to starch highly increases both the elastic modulus and the tensile strength and reduces the elongation at break of the blends. This fact agrees with the increase in the interaction effect due to the hydrogen interaction between the polymeric chains. As expected, the addition of glycerol into raw materials promotes a better movement of the polymer chains, resulting in a strong increase in the elongation at break. However, as previously reported in the bibliography [44], in the case of starch/gelatin blends, the formation of an ordered structure by intermolecular hydrogen bonding promotes rigidity. Additionally, phase separation shown in the SEM images, resulting in the formation of small starch regions, can affect the mechanical response. This increase in the elasticity may be attributed to the increasing interactions between the hydroxyl groups present in glycerol and the carboxyl groups found in amylose in starch and protein in gelatin. The minor addition of starch has been shown to enhance the interfacial interactions between gelatin and starch within the blend [45].

### 3.7. Water Vapor Transmission Rate (WVTR)

**Figure 8:**
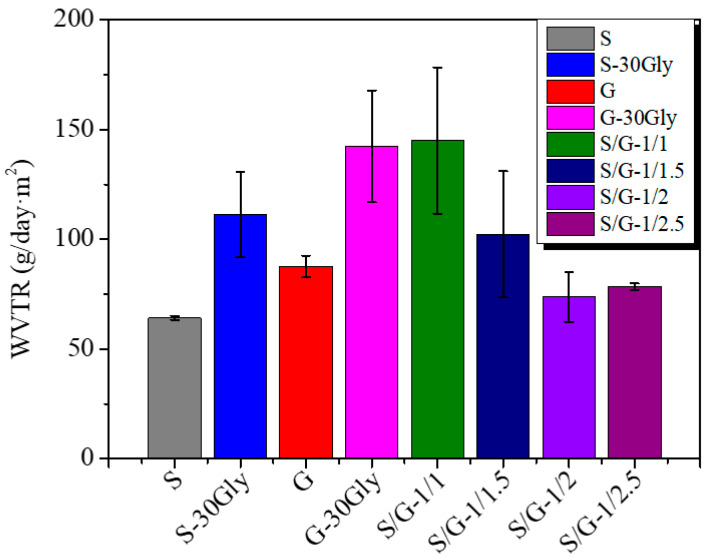
Water vapor transmission rate values for all studied samples normalized to 100 microns.

The water vapor barrier performance of the developed blends is characterized by the water vapor transmission rate (WVTR). These results are shown in Figure 8. As is known, a controlled WVTR number signifies that water vapor will have difficulty passing through, thereby extending the shelf life of the product, i.e., food, inside the packaging [46]. This value may vary depending on the nature of the food [47].

In our case, an increase in the WVTR values when comparing S and G samples is obtained, as reported in Figure 8. A comparison of the WVTR of neat samples with their plasticized counterparts reveals an increase in the former’s value, as expected in a plasticized system. This is due to an increased polymer chain mobility [48,49], increasing the water diffusion process and, therefore, the WVTR of these samples [50].

In the case of starch/gelatin blends, the WVTR tends to decrease with the increase in gelatin. In particular, a WVTR value similar to the value obtained by Channa et al. [38]. In their study, a blend of starch/gelatin with a ratio of 1/2 was prepared and plasticized with glycerol, obtaining a WVTR value of approximately 80 g/day·m^2^.

### 3.8. Surface Roughness

Surface roughness measures have been demonstrated to provide information regarding the tomographic profile of the samples. The behavior of a material in contact with solvents is determined by a combination of chemical composition and tomography. An increase in surface roughness has been demonstrated to result in a decrease in surface compatibility with solvents, such as water, leading to an increase in the value of the angle observed in WCA measures. The analysis has been conducted on four distinct points from each film. The results of this analysis are presented in Table 4.

In neat samples, Sa (arithmetical mean height over an area) values approach 0, suggesting a high degree of homogeneity and a negligible presence of roughness compared to other polymers, such as PLA (0.88 µm) and PC (0.45 µm) [51]. Non-blended samples show low roughness, except for the neat starch, in which the addition of the plasticizer greatly improves the surface homogeneity. However, the addition of gelatin to the sample results in a shift in this value, leading to an increase in the roughness until it reaches its maximum in the S/G-1/2.5 sample, which contains a higher quantity of gelatin. This difference may be explained through a phase separation between both polymers with an increase in the crystallinity of one of the phases, as demonstrated in the case of low starch concentrations by Podshivalov et al. [45] in potato starch and gelatin edible films.

Height ratio is a quantitative metric that quantifies the disparity between the maximum and minimum surface elevations. This ratio is also associated with the surface roughness and its interaction with solvent droplets, such as water. As observed, the results obtained exhibited a behavioral pattern analogous to that observed in Sa; it increases with gelatin content, reaching its maximum with the highest amount of gelatin, S/G-1/2.5. As a result, the surface hydrophilicity of the polymeric blends is expected to decrease as the gelatin content increases.

### 3.9. Water Contact Angle (WCA)

**Figure 9:**
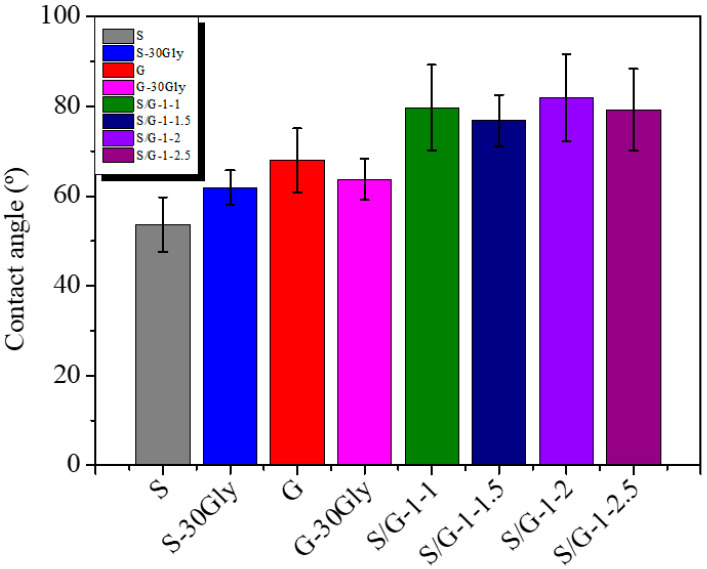
Water contact angle values for all the studied samples.

The wettability of the films is determined by water contact angle measurements, compiled in Figure 9.

The values observed on gelatin films can be attributed to the hydrophilic amino acids in the matrix, which promote interaction between water molecules and the matrix, helping to create a hydrophilic surface, as previously reported in the scientific literature [52]. As the starch/gelatin ratio changed, the WCA increased from 54° for neat starch and 68° for neat gelatin up to 82° for the blend of starch/gelatin ratio of 1–2. This indicates a shift from a starting hydrophilic behavior to a slightly more hydrophobic one. Also in this case, the interaction between starch, glycerol, and gelatin may promote the formation of a network with the interaction effect due to the hydrogen interaction between the polymeric chains decreasing the available hydrogen bonds, showing scattered values, this can be explained due to the increase in the surface roughness related to a higher degree of interactions as the gelatin content increases [53].

### 3.10. Moisture Absorption and Moisture Loss

**Figure 10:**
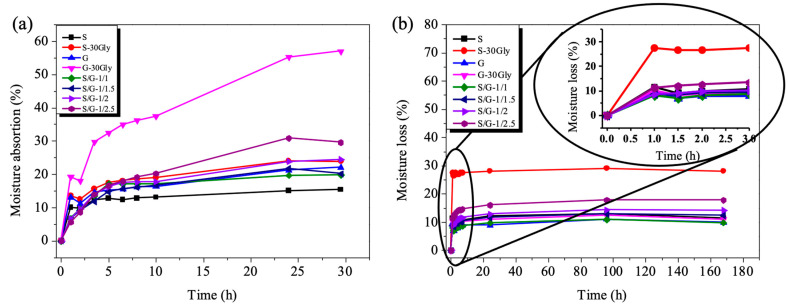
Moisture behavior of the samples: moisture absorption (**a**) and moisture loss (**b**).

The dependence of both moisture absorption and moisture loss with time at room temperature, shown in Figure 10, was studied to determine the time required to activate both the programming and recovery steps for the humidity/mechanical cycles used to evaluate the humidity-activated shape-memory behavior of the plasticized starch/gelatin blends.

For all the plasticized starch/gelatin blends, Figure 10a,b illustrates the time dependence of moisture absorption (essential to recover the original shape) and of moisture loss (essential for the temporary shape fixation), respectively.

Regarding moisture absorption, as shown in Figure 10a, all samples tend to reach an equilibrium state after 2 h, except for the sample with gelatin with 30 wt% glycerol, which exhibits two absorption phases: the first phase occurs from the beginning of the experiment to 2 h, and the second phase spans from 2 h until the end of the measurements. Additionally, the sample with a starch/gelatin ratio of 1/2.5 reaches equilibrium after 5 h from the start of the experiment.

In Figure 10b, a consistent slope of the moisture loss curves is observed across all analyzed samples, indicating that all the starch/gelatin blends lost moisture very quickly within the first 1.5 h. Additionally, for all the starch/gelatin blends, the slope of moisture loss (Figure 10b) was significantly steeper than that of moisture absorption (Figure 10a), indicating a faster rate of moisture loss. Considering the observed results, it is clear that the activation process begins approximately one hour after the start.

### 3.11. Water Uptake Rate (WU)

**Figure 11:**
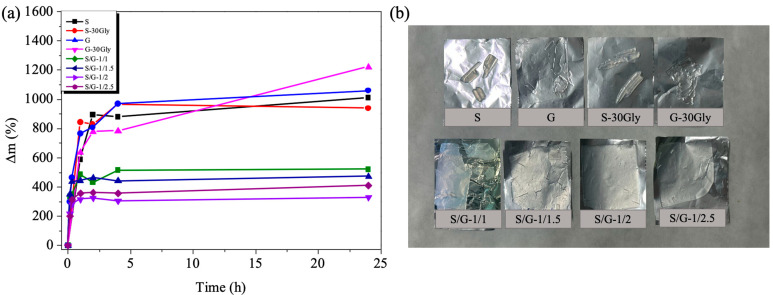
(**a**) Water uptake of the studied samples, (**b**) visual appearance of the films after 24 h of immersion time.

Upon water immersion, a hydrophilic polymer undergoes a process of liquid molecule diffusion, resulting in material swelling. Results obtained upon immersion in water for 24 h, as well as the visual appearance of the samples, are presented in Figure 11.

The water uptake increased with the immersion time for times lower than 2 h, as shown in Figure 11a. Neat samples, as expected, absorbed a very high content of water, twice that of their blends. Moreover, blends presented a significantly lower swelling value compared with neat samples. In the case of blend formulations, the decrease in the swelling degree may be due to the restricted chain movement as a consequence of the interactions between both polymers, showing also higher stability without losing its initial shape, Figure 11b.

### 3.12. Humidity-Activated Shape-Memory Behaviour

Consequently, one hour was designated for studying the humidity-activated shape-memory response exhibited by these starch/gelatin blends at about 20 °C.

**Figure 12:**
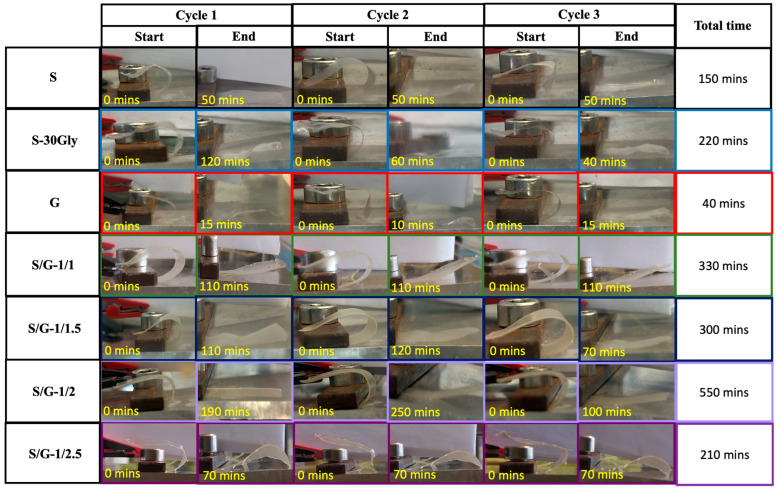
Visual appearance of the films through the humidity-activated shape-memory study.

In particular, for the programming stage, the sample is conditioned for 1 h at a relative humidity of 72%. After bending the sample (Figure 1), the sample is dried at a relative humidity of 10% for one hour while maintaining constant bending to fix the temporary shape. Then, in the recovery stage, the bending forces on the film are released in dry conditions and the sample is inserted into the humidity chamber (RH = 72%) to recover its original shape, Figure 12 shows the angle-based shape-memory evolution based on the visual appearance of the films through all the cycles performed as well as the duration needed to recover the original shape in each cycle.

**Figure 13:**
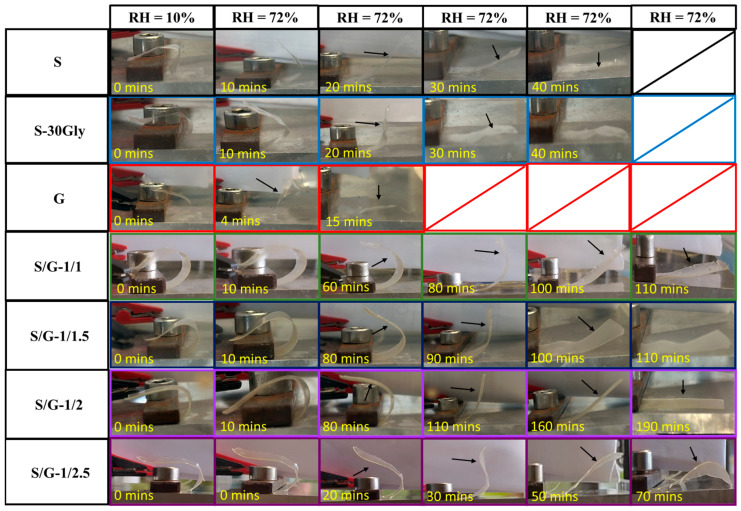
The first cycle of the humidity-activated shape-memory for the studied samples. The arrows help to visualize the samples due to their transparency.

A visual result of the humidity-activated shape-memory recovery step for each sample is shown in Figure 13.

The calculated shape recovery ratio and shape fixity ratio for each cycle and each sample are reported in Table 5.

As shown in Figure 12, all samples were able to recover their initial structure from the programmed temporal shape, presenting high ratios for both shape recovery and shape fixity, as shown in Table 5. In particular, starch and starch/gelatin blends show excellent fixity values with a shape fixity ratio of 100%, while G sample and S/G-1/2.5 show values smaller than 100%.

The strain recovery ratios for all the samples are very high, higher than 90% in all cases. The fastest sample to recover its original shape is G, at 40 min. Neat starch is quite fast, at about 150 min, while all the blends need more than 300 min to complete the first humidity-activated shape-memory cycle. At the highest amount of gelatin in the blend, S/G-1/2.5, only 210 min are necessary to recover the original shape of the sample.

In general, an increased amount of gelatin requires a longer time to recover the original shape. This fact can be attributed to the -OH groups presented in all the materials, starch, glycerol and gelatin and their interaction, thus considering that the % between starch and glycerol is fixed to 30 and the gelatin is added in different amounts with respect to starch, varying the % of -OH free in the blend.

## 4. Conclusions

In this work, both neat and plasticized starch as well as gelatin and their blends are studied, taking into account the effect of the different amounts of gelatin on the final behavior of the materials. Thus, considering their potential application as food packaging, the mechanical response, as well as wettability, water permeability, and water uptake rate, is studied. In particular, in terms of mechanical properties, as expected, the plasticizer effect strongly affects the mechanical response of both starch and gelatin, leading to blends with values in between neat and plasticized materials. However, a clear reinforcing effect in terms of tensile strength is found in the blend with the highest amount of gelatin, (S/G-1/2.5), showing the highest tensile strength. Wettability of the blends also increased with respect to the neat materials, while maintaining, however, their hydrophilic behavior. The result is different for water vapor permeability, which is driven by the plasticizer effect more than the addition of different amounts of gelatin into the blends. The water uptake increased with the immersion time for times lower than 2 h, for all the samples; however, blends presented a significantly lower swelling value compared with the neat samples. Therefore, in this work, the strong moisture dependence of both polymers has been studied as a potential advantage and not a negative point, as is usually considered. In particular, the capability of starch as well as of plasticized starch to show humidity-activated shape-memory behavior has been studied and confirmed in this paper. To the best of our knowledge, the humidity-activated shape-memory effect of gelatin, as well as in plasticized gelatin and starch/gelatin blends, is studied in this work for the first time, studying the effect of the addition of gelatin in different amounts to the plasticized starch. In all the cases, starch and gelatin, both neat and plasticized, as well as their plasticized blends, show excellent values in terms of both strain fixity ratio and strain recovery ratio. In particular, showing values of *R_f_* of about 100% in all cases and *R_r_* with values higher than 90% for the samples studied.

## Figures and Tables

**Table 1:** Percentage of each component in each of the studied samples.

Sample	Composition (w%)		
	Starch	Glycerol	Gelatin
S	100	-	-
S-30Gly	70	30	-
G	100	-	-
G-30Gly	-	30	70
S/G-1/1	43.5	13	43.5
S/G-1/1.5	35.7	10.7	53.6
S/G-1/2	30.3	9.1	60.6
S/G-1/2.5	26.3	7.9	65.8

**Table 2:** Maximum degradation temperature (T_max_) of the different samples.

Sample	T_max_ (°C)
S	291
S-30Gly	297
G	301
G-30Gly	289
S/G-1/1	289
S/G-1/1.5	288
S/G-1/2	288
S/G-1/2.5	291

**Table 3:** Summary of the mechanical properties for all the samples. Different letters in the column indicate significant differences according to Tukey’s test (*p* < 0.05). * Values are significant at *p* < 0.05.

Sample	E (MPa)	σ (MPa)	ε (%)
S	1951 ± 134 ^a^	53 ± 1 ^a^	9 ± 2 ^a^
S-30Gly	507 ± 105 ^b^	11 ± 2 ^b^	44 ± 9 ^b^
G	1213 ± 118 ^c^	73 ± 16 ^a^	17 ± 7 ^a^
G-30Gly	693 ± 181 ^b^	27 ± 2 ^b^	71 ± 12 ^c^
S/G-1/1	1326 ± 160 ^c^	45 ± 9 ^c^	11 ± 2 ^a^
S/G-1/1.5	945 ± 162 ^b, c^	47 ± 17 ^c^	10 ± 2 ^a^
S/G-1/2	1090 ± 312 ^c^	56 ± 13 ^d^	10 ± 1 ^a^
S/G-1/2.5	1361 ± 61 ^c^	66 ± 7 ^d^	23 ± 2 ^a^
F ratio	2.5543	8.30623	3.63972
*p*-Value	0.03224 *	0.0000083 *	0.00766 *

**Table 4:** Values of arithmetical mean height over an area (Sa) and height ratio for all studied samples.

Sample	Sa (µm)	Height Ratio
S	1.2 ± 0.1	1.49 ± 0.03
S-30Gly	0.36 ± 0.06	1.183 ± 0.005
G	0.35 ± 0.03	1.19 ± 0.01
G-30Gly	0.32 ± 0.01	1.198 ± 0.003
S/G-1/1	1.4 ± 0.3	1.87 ± 0.06
S/G-1/1.5	2.5 ± 0.3	1.93 ± 0.07
S/G-1/2	2.5 ± 0.5	1.52 ± 0.02
S/G-1/2.5	4.0 ± 0.2	1.62 ± 0.06

**Table 5:** Shape recovery ratio (*R_r_*) and shape fixity ratio (*R_f_*) for each shape-memory cycle for each sample.

Sample	Cycle	*R_f_* (%)	*R_r_* (%)
S	1	100	97.6
	2	100	96.6
	3	100	99.0
S-30Gly	1	100	94.9
	2	100	96.8
	3	100	99.1
G	1	85.2	99.1
	2	94.5	96.5
	3	100	91.2
S/G-1/1	1	100	94.8
	2	100	89.4
	3	100	92.1
S/G-1/1.5	1	100	91.5
	2	100	96.0
	3	100	94.0
S/G-1/2	1	100	99.7
	2	100	93.2
	3	100	97.9
S/G-1/2.5	1	95.8	97.9
	2	88.9	94.1
	3	89.5	97.2

## Data Availability

The original contributions presented in this study are included in the article. Further inquiries can be directed to the corresponding author.
